# Stochastic spontaneous calcium release events and sodium channelopathies promote ventricular arrhythmias

**DOI:** 10.1063/1.4999612

**Published:** 2017-08-23

**Authors:** Fernando O. Campos, Yohannes Shiferaw, Edward J. Vigmond, Gernot Plank

**Affiliations:** 1 Department of Congenital Heart Diseases and Pediatric Cardiology, German Heart Institute Berlin, Berlin, Germany; 2 Institute of Biophysics, Medical University of Graz, Graz, Austria; 3 Department of Physics, California State University, Northridge, California 91330, USA; 4 LIRYC Institute, University of Bordeaux, Bordeaux, France; 5 Department of Electrical and Computer Engineering, University of Calgary, Calgary, Alberta, T2N 1N4, Canada

## Abstract

Premature ventricular complexes (PVCs), the first initiating beats of a variety of cardiac arrhythmias, have been associated with spontaneous calcium release (SCR) events at the cell level. However, the mechanisms underlying the degeneration of such PVCs into arrhythmias are not fully understood. The objective of this study was to investigate the conditions under which SCR-mediated PVCs can lead to ventricular arrhythmias. In particular, we sought to determine whether sodium (Na^+^) current loss-of-function in the structurally normal ventricles provides a substrate for unidirectional conduction block and reentry initiated by SCR-mediated PVCs. To achieve this goal, a stochastic model of SCR was incorporated into an anatomically accurate compute model of the rabbit ventricles with the His-Purkinje system (HPS). Simulations with reduced Na^+^ current due to a negative-shift in the steady-state channel inactivation showed that SCR-mediated delayed afterdepolarizations led to PVC formation in the HPS, where the electrotonic load was lower, conduction block, and reentry in the 3D myocardium. Moreover, arrhythmia initiation was only possible when intrinsic electrophysiological heterogeneity in action potential within the ventricles was present. In conclusion, while benign in healthy individuals SCR-mediated PVCs can lead to life-threatening ventricular arrhythmias when combined with Na^+^ channelopathies.

Cardiovascular diseases, primarily due to ventricular arrhythmias, continue to be the leading cause of sudden death in the industrialized world. Reentrant activations are believed to be implicated with clinically relevant arrhythmias. Reentry results from a combined effect of triggered activity and unidirectional conduction block. It is well established that triggered activity precipitating premature ventricular complexes (PVCs) are a consequence of spontaneous calcium (Ca^2+^) release (SCR) events at the subcellular level. However, the factors determining whether these PVCs will degenerate into reentry are not as well understood. This study makes use of a state of the art computational model of the rabbit ventricles and His-Purkinje system (HPS) to investigate how organ-scale arrhythmias emerge from abnormal SCR events at the subcellular level. Simulation results demonstrate that reduced sodium (Na^+^) channel availability promotes arrhythmia formation by SCR-mediated PVCs in the structurally normal heart.

## I. INTRODUCTION

Ventricular arrhythmias precipitating cardiac arrest are considered the most common mechanisms of sudden death. Despite intense research and debate over the past decades, the precise mechanisms underlying the formation of arrhythmias remain incompletely understood. It is assumed that reentrant activations play a pivotal role in maintaining many clinically important ventricular arrhythmias and that their initiation is the result of the combined effect of triggered activity and unidirectional conduction block.^1^

Triggered activity in cardiac myocytes occurs when action potentials (APs) are elicited by oscillations in the transmembrane potential, *V_m_*, referred to as afterdepolarizations, which are assumed to be caused by abnormalities at the subcellular scale. Such afterdepolarizations may manifest either early during the plateau phase of the AP – classified then as early afterdepolarizations (EADs) – or later, after the AP has repolarized to resting potential levels, in which case they are referred to as delayed afterdepolarizations (DADs).^2^ DADs can be classified as subthreshold or suprathreshold depending on whether the Na^+^ channels are reactivated. Their formation has been associated with disturbances in the subcellular Ca^2+^ cycling.^3–5^ During the course of an AP, Ca^2+^ sparks arise throughout the cell due to the opening of Ryanodine receptor (RyR) channels located in the sarcoplasmic reticulum (SR) within the dyadic clefts forming a Ca^2+^ release unit. The summation of these coordinated Ca^2+^ sparks over the whole cell forms the Ca^2+^ transient that regulates contraction.^4^ However, under certain conditions, Ca^2+^ release from the SR can emerge spontaneously due to stochastic RyR fluctuations that occur independent of an AP. Such random RyR openings can induce spontaneous Ca^2+^ sparks, which propagate between adjacent dyadic spaces to form Ca^2+^ waves. These spontaneous Ca^2+^ release events cause an increase in the subcellular Ca^2+^ concentration, which in turn stimulates Ca^2+^-sensitive inward currents leading to a DAD.^4^ SCR events are promoted by conditions producing Ca^2+^ SR overload and/or enhancing basal RyR channel activity, such as ischemia,^6^ heart failure (HF),^7^ and catecholaminergic polymorphic ventricular tachycardia (CPVT).^8^ Notwithstanding that SCR-mediated DADs can lead to triggered APs in the isolated cell, this is not necessarily the case in well-coupled cardiac tissue. A myocyte eliciting a DAD will cause current to flow downstream to its quiescent neighbors through gap junctions. Thus less current is available for depolarizing the local membrane which reduces the DAD amplitude. In the ventricles, a large number of adjacent myocytes must undergo a DAD with sufficient temporal synchronization to achieve a suprathreshold depolarization over a liminal region to drive a propagated response.^9^ A mechanism by which DADs can summate to overcome electrotonic load and cause a PVC has been proposed by our group in a recent modeling study^10^ wherein we demonstrated that Ca^2+^ overload and concomitantly increased SR load reduce the timing variability of SCR events, thus providing an implicit synchronization mechanism for achieving the necessary degree of coincidence of DADs across cells to trigger a PVC.

In the vast majority of cases PVCs are innocent, triggering only an isolated ectopic heartbeat.^11^ However, in rare cases propagation of a PVC may be blocked unidirectionally and reentry may be induced. Propagated PVCs can be blocked due to heterogeneities in the tissue's effective refractory period (ERP) or due to a local reduction of the safety factor of propagation caused by the a mismatch between current provided by diffusion and the local current required to depolarize the membrane to threshold.^12^ The ERP is the period during which myocytes cannot generate new APs since the fast Na^+^ channels are not yet fully recovered from inactivation. The ERP is slightly shorter than the AP duration (APD) and serves as a protective mechanism by preventing an overly fast recurrent activation of tissue and thus the return of a depolarization wavefront to its own site of origin. In addition to AP generation, Na^+^ channels also play an important role in impulse conduction in cardiac tissue. Impaired excitability due to reduction of the fast Na^+^ current can exacerbate local source-sink mismatch, another substrate for conduction block of PVCs.^13^ Subthreshold DADs have been recently demonstrated as being capable of rendering simple 1D and 2D cardiac tissues prone to conduction block.^14^ However, the factors setting the stage for the development of DAD-related arrhythmias in the ventricles with heterogeneous electrophysiological properties, anatomical structures with complex fiber orientations and the presence of the HPS are not as well established.

The present study builds upon our previous work with an anatomically realistic model of the rabbit ventricles and HPS^10^ to investigate the conditions under which PVCs caused by stochastic SCR events can degenerate into arrhythmias. Particularly whether Na^+^ current loss-of-function mimicking acquired and inherited cardiac diseases, such as the HF^15^ and Brugada syndrome,^16^ can promote conduction block and reentry in the structurally healthy ventricles. We test the hypotheses that (1) reduced Na^+^ current accentuates the occurrence of subthreshold DADs in the 3D myocardium causing conduction block of PVCs; and (2) electrophysiological heterogeneity in APD in ventricular cells is necessary for initiation of arrhythmias induced by SCR-mediated PVCs.

## II. METHODS

We have developed a multiscale computational model of the rabbit ventricles equipped with a topologically realistic model of the HPS for use in electrophysiological studies. The major components of the model are outlined below.

### A. Rabbit biventricular model

#### 1. Ventricles

**FIG. 1.**
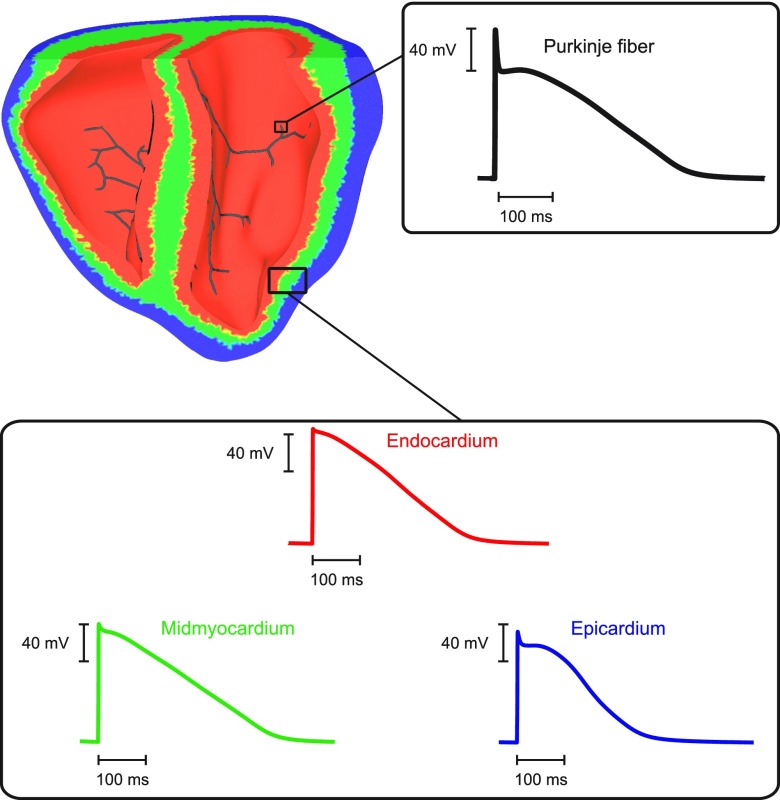
Assignment of transmural regions in the rabbit BiV model: endocardial (red), midmyocardial (green), and epicardial (green) layers. Insets: single cell APs from DAD-prone Purkinje (black line), endocardial (red line), midmyocardial (green line), and epicardial (blue line) cells.

Electrical activity was simulated within a tetrahedral finite element model of the rabbit ventricles.^17^ The model is a refined version of the San Diego rabbit model, which represents the gross biventricular (BiV) geometry and includes the anatomically realistic fiber architecture.^18^ Briefly, the model contains 547 680 myocardial nodes defining 3 073 529 tetrahedral elements with a mean discretization of 279 *μ*m. Two versions of the BiV model were used in this study: a homogeneous model in which the same electrophysiological properties were considered in ventricles as well as in the HPS, and a heterogeneous model. Intrinsic electrophysiological heterogeneity in the ventricles is incorporated into the BiV model based on previous modeling studies.^19,20^ Specifically, the ventricles were divided into three transmural regions: endocardium (30%), midmyocardium (40%), and epicardium (30%), as well as apex-to-base regions: apex, mid, and base. This resulted in nine distinct regions, to each of which ionic models with distinct cellular dynamics were assigned as shown in Fig. 1.

#### 2. His-Purkinje system

The BiV model in this study is equipped with a topologically realistic model of the HPS. The HPS topology is a simplified representation of the real physiological system (see Fig. 1). It is based on anatomical observations such as the arborization pattern in the left (LV) and right (RV) ventricles representing the anterior, posterior, and septal fascicles in the LV as well as two distinct fascicles in the RV. It also reproduces major bifurcation points in the left and right subsystems. Electrical activity was simulated within one-dimensional cubic Hermite elements (2232 segments) separated by discrete gap junctions that are modeled as fixed resistances.^21^ Constraints imposed upon the system ensured current continuity between segments and conservation of current at bifurcations. At each endpoint of the HPS, a Purkinje node (Purkinje-ventricular junction—PVJ) is coupled to myocardial nodes by a fixed resistance to account for the experimentally observed asymmetry in conduction delays across PVJs. Generation of the BiV model and the incorporation of the HPS network have been described in detail elsewhere.^17^

### B. Model of spontaneous Ca^2+^ release and action potential

In cardiac myocytes, subcellular Ca^2+^ and voltage dynamics are typically modeled by a system of non-linear ordinary differential equations. Over the past years, biophysically detailed models accounting for the stochastic and spatiotemporal features Ca^2+^ spark formation have been developed.^22–24^ However, the use of such highly detailed models in organ scale simulations are computationally prohibitive. In this work, we make use of a phenomenological model accounting for experimentally observed features of spontaneous Ca^2+^ release (SCR) events. A SCR event is represented in the model as a Ca^2+^ wave that is nucleated in the cell and then propagating in a fire-diffuse-fire way. SCR events in our model have a functional dependence on SR Ca^2+^ load (Ca_*SR*_), which ensures that SCR events are more likely to occur as the SR becomes overloaded. Further details on how Ca^2+^ nucleation is modeled can be found elsewhere.^25^ The phenomenological model of SCR events was coupled to the Mahajan–Shiferaw (MSH)^26^ rabbit ventricular AP model and used to simulate cellular dynamics in both HPS and ventricles.

#### 1. Electrophysiological heterogeneity

##### a. Purkinje fibers

Individual ionic currents of the MSH model were modified to represent intrinsic electrical properties of Purkinje fibers.^27^ Accordingly, maximum conductances of the fast Na^+^ current (*g_Na_*), the rapid (*g_Kr_*), and slow (*g_Ks_*) delayed-rectifier potassium currents as well as the extracellular potassium concentration (*K_e_*) were adjusted to reproduce the main features of Purkinje AP (e.g., upstroke velocity, APD and resting potential). Table I summarizes the modifications made to the MSH model to reproduce the Purkinje AP.

**TABLE I.** Relative adjustments made to default parameters of the MSH model.

Parameter	PF^a^	Endo	Mid	Epi
*g_*Na*_*	1.6	0.8	0.9	0.7
*g_*tof*_*	1.0	0.01	0.1	1.0
*g_*tos*_*	1.0	2.0	1.5	1.0
*g_*Kr*_*	0.5	1.0	0.5	1.5
*g_*Ks*_*	0.3	1.0	0.8	2.5
*K_*e*_*	1.15	1.0	1.0	1.0

^a^ PF, Endo, Mid and Epi: Purkinje fiber, endocardium, midmyocardium, and epicardium.

##### b. Ventricular myocytes

The MSH model was also modified to represent electrophysiological heterogeneities known to exist in the ventricles. Specifically, maximum conductivities of the fast transient-outward current (*g_tof_* and *g_tos_*), *g_Na_*, *g_Kr_*, and *g_Ks_* in the MSH model were adjusted to make up the intrinsic electrophysiological differences in AP shape and duration of endocardial, midmyocardial, and epicardial myocytes.^19,28^ Table I lists the above-mentioned modifications made to the MSH model. Furthermore, in rabbits, the APD is longer at the apex than at the base.^19^ To incorporate this characteristic into the BiV model, *g_Ks_* was rescaled by factors of 2.0, 1.5, and 1.0 in the base, mid, and apex regions, respectively.

##### c. DAD-prone model

To generate DADs, key parameters of the MSH model were modified.^25^ In line with experimental evidence showing that electrophysiological remodeling under pathologies, such as HF^7^ increases the propensity for DADs, the strength of the electrogenic sodium-calcium exchange current (*I_NCX_*) was doubled and the maximum conductance of the inward rectifier potassium current ($g_{K1}$) was decreased to 30% of its control value. Moreover, the spontaneous SR Ca^2+^ release strength *g_sp_* in Purkinje fibers was increased by 50%. If not stated otherwise, these values were used throughout the study.

##### d. Reduced Na^+^

Simulations were conducted under two electrophysiological conditions representing different forms of global fast Na^+^ current loss-of-function: reducing *g_Na_* to 75%, 50%, and 25% of values in Table I); and shifting the steady-state curves of the Na^+^ channel inactivation gating variables *h* and *j* in the negative direction, i.e., by −5 mV (Ref. 14) to account for electrophysiological changes associated with ischemia,^29^ HF^15^ and long Q-T (LQT3) and Brugada syndromes.^30,31^

### C. Governing equations

Electrical activity within the BiV model was simulated using the monodomain equations expressed as

$$\nabla \cdot (\sigma_{m}\nabla V_{m})=\beta I_{m},$$ (1)

$$C_{m}\frac{\partial V_{m}}{\partial t}+I_{ion}(V_{m},\eta )-I_{\text{stim}}=I_{m},$$ (2)

$$\frac{\partial \eta }{\partial t}=f(V_{m},\eta ),$$ (3)

where $\sigma_{m}=\text{diag}(\sigma_{ml},\sigma_{mt},\sigma_{mt})$ is the harmonic mean conductivity tensor or the effective bulk conductivity;^32^ *V_m_* is the transmembrane voltage; *β* is the surface to volume ratio; *I_m_* is the transmembrane current density; *C_m_* is the membrane capacitance per unit area; *I_ion_* is the density of the total ionic current flowing through the membrane channels, pumps and exchangers; and *I_stim_* is the stimulus current density. *I_ion_* depends on *V_m_* as well as on a set of state variables η which describes channel gating and ionic concentrations according to the vector-valued function $f(V_{m},\eta )$. See the MSH model^26^ for further details about *I_ion_*, η, and $f(V_{m},\eta )$.

The intracellular domain, comprising the cardiac tissue, was modeled with conductivity values of *σ_ml_* = 0.14 S/m and *σ_mt_* = 0.018 S/m.^33^ The bath-loading effects using these values have been demonstrated to be very minor and thus were neglected.^32^

Solutions to the monodomain Eqs. (1)–(3) were performed using the Cardiac Arrhythmia Research Package (CARP).^34^ The underlying numerical techniques to solve the discretized linear systems of equations have been described in great detail previously.^35,36^

### D. Simulation protocol

#### 1. Single-cell simulations

The DAD-prone MSH myocyte models were paced at 2.0 Hz for 100 cycles to stabilize under different extracellular Ca^2+^ concentrations Ca_*e*_. The pacing procedure was repeated iteratively with varying Ca_*e*_ until a prescribed Ca_*SR*_ of 1600 *μ*mol/l was achieved at the end of the pacing protocol. Once the models have stabilized, a 1000-ms pause was simulated in order to see whether DADs and/or triggered APs would emerge. Such constant train of stimulations followed by a halt in pacing is a widely used protocol to assess for the presence of SCR events and triggered activity.^8,37^ Further details about the pacing protocol can be found in our previous study.^10^

#### 2. Tissue simulations

Unlike single cells, pacing organ scale models for 100 cycles is computationally expensive. To save computational efforts, single-cell model states (e.g., *V_m_*, Ca_*SR*_, channel gating variables, etc.) at the end of the pacing protocol were stored and used to initialize the BiV model. This initialization procedure is equivalent to pacing the entire tissue model in a space-clamped mode. Initial states were the same for cells belonging to each region of the BiV model (e.g., HPS, basal endocardium, apical epicardium, etc.). Moreover, due to the stochastic nature of the SCR events, N = 100 simulations for each set of experiments were performed. Different seeds were assigned to each individual cell in the BiV model to ensure heterogeneous stochastic SCR evolution.^10^

##### a. Heterogenous Ca_*SR*_ distribution

Strength as well as waiting time of SCR events in our phenomenological cell model depend on Ca_*SR*_. The average waiting time of SCR events in our phenomenological model is 142 ms ± 50 ms for a Ca_*SR*_ of 1600 *μ*mol/l.^10^ In order to widen strength and timing variability of SCR events, Ca_*SR*_ values drawn from a standard uniform distribution on the interval [1500 *μ*mol/l, 1700 *μ*mol/l] were generated and assigned to each cell in the BiV model.

### E. Data analysis

In all experiments, the transmembrane voltage *V_m_* was recorded over the 1000-ms pacing pause. The number of simulations in which a PVC was observed, *n*, was recorded to compute the probability $p_{PVC}=n/N$ during the pacing pause. PVCs that did not activate more than 10% of the ventricles were not computed in *p_PVC_*. PVCs activating more than 50% of the ventricles were considered propagated PVCs while PVCs that failed to do so were classified as block. The probability of a reentry due to a PVC was also calculated. A reentry lasting for more than 1000 ms is considered to be sustained. In addition, the waiting times until the onset of a PVC, *T_PVC_*, was determined as the time elapsed from the end of pacing until the instant of a triggered AP crossing −10 mV. APD was calculated as the difference between *T_PVC_* and the repolarization time (time at which the AP waveform crossed a level corresponding to 90% of repolarization to the resting potential). Finally, the location of the first PVC was also recorded.

## III. RESULTS

### A. SCR-mediated APs and subthreshold DADs in isolated myocytes

**FIG. 2.**
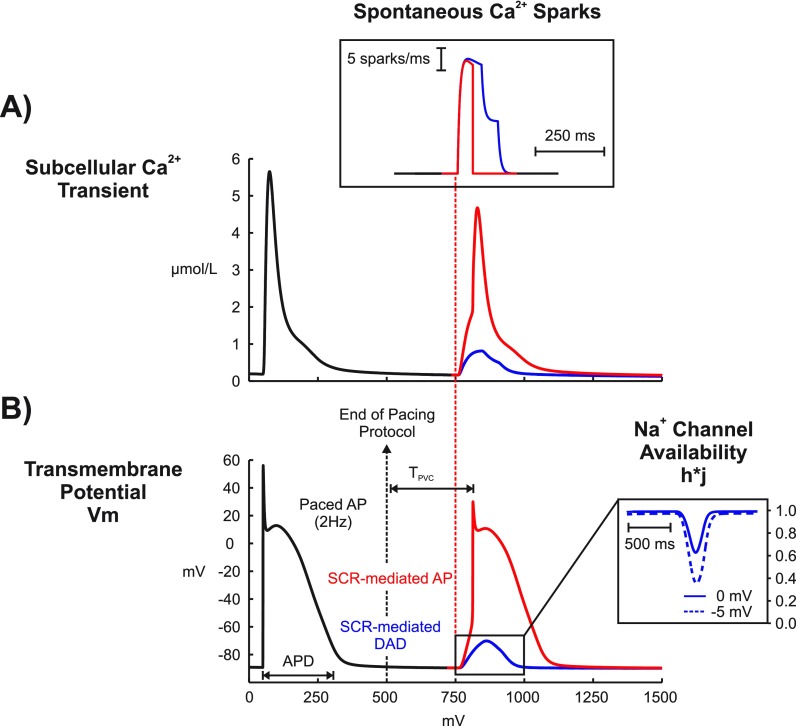
SCR-mediated triggered activity following a paced AP in an isolated DAD-prone myocyte. (a) Subcellular Ca^2+^ transients resulting from a paced AP (black trace) and from two different stochastic SCR (inset) events (blue and red traces) originating approximately at time t = 750 ms. (b) *V_m_* traces of the last paced AP (out of 100 pacing cycles) before a 1000-ms pause. Traces taken from the same two experiments in (a). The elevations in the subcellular Ca^2+^ induced membrane depolarizations which either turned into a subthreshold DAD (blue trace) or a full-blown AP (red trace). Inset: h*j evolution for the subthreshold DAD (solid line) in (a) and for the same experiment with a 5-mV negative-shift in the steady-state Na^+^ channel inactivation (dashed line).

The augmented MSH cell model was paced for 100 cycles according to the protocol previously described. Once the model has stabilized, a 1000-ms pause was simulated and changes in *V_m_* were tracked to see whether DADs and/or triggered APs due to SCR events emerge. Figure 2 illustrates two different results obtained with our stochastic SCR model (only the last paced beat is shown - black trace). In both simulations, Ca^2+^ sparks due to SCR events (inset) led to increases in the subcellular Ca^2+^ concentration [Fig. 2(a) that either caused a subthreshold DAD (blue trace) or an AP (red trace) in Fig. 2(b). As can be seen in the inset of Fig. 2(b), the DAD led to a decrease in Na^+^ channel availability (h*j). The exact same experiment causing the subthreshold DAD was repeated the with a 5-mV negative-shift in the steady-state Na^+^ channel inactivation (dashed). Note that the negative-shift led to a larger reduction in h*j.

### B. SCR-mediated PVCs and reentry

In accordance with the protocol described above, 5 × 100 distinct simulations (control, *g_Na_* reduced by 25%, 50%, and 75% and negative shifting the Na^+^ channel steady-state inactivation) were conducted for both the homogeneous and the heterogeneous BiV model and monitored for spontaneous PVCs and electrical reentries.

#### 1. Homogeneous BiV model

SCR-mediated PVCs were observed in all (N = 100) control experiments with the homogeneous BiV Model. The average *T_PVC_* was 187 ± 10 ms. All foci arose exclusively in the HPS and no reentries were detected. Reducing *g_Na_* to 75% of its control value changed neither *p_PVC_* nor the location of the foci. Further *g_Na_* reduction to 50% did not change *p_PVC_*, but the location of the foci was distributed in both HPS (60%) and myocardium (40%). PVCs were detected in only 2 out of N = 100 simulations with severe *g_Na_* reduction to 25%. They appeared in the HPS and blocked in the ventricles. *T_PVC_* increased to 192 ± 10 ms, 205 ± 12 ms, and 235 ± 1 ms in experiments with *g_Na_* decreased to 75%, 50%, and 25%, respectively. No reentrant episodes were observed in any of the experiments performed. With a negative-shift in the steady-state Na^+^ channel no PVCs were inactivated.

#### 2. Heterogeneous BiV model

In simulations with the heterogeneous BiV model, SCR-mediated PVCs were observed in all control experiments with average *T_PVC_* = 161 ± 9 ms. All foci appeared in the HPS and no reentries were detected. Similar to the homogeneous case, *g_Na_* reduction did not affect *p_PVC_* or location of the foci. *T_PVC_* increased to 168 ± 9 ms, 174 ± 9 ms, and to 203 ± 12 ms in simulations where *g_Na_* was reduced to 75%, 50%, and 25% of its control value, respectively. Reentries were observed in two simulations with *g_Na_* reduced to 25%, but self-terminated within <1000 ms.

Reduced Na^+^ due to a negative-shift in the steady-state Na^+^ channel inactivation had a major effect in PVC propagation and arrhythmia induction. PVCs were observed in all simulations with the majority of foci (83 out of N = 100) emerging in the HPS. However, 17% of these PVCs did not propagate throughout the ventricles. Average *T_PVC_* was similar to *g_Na_* reduction to 25%: 202 ± 19 ms, but blocked PVCs emerged later at *T_PVC_* = 230 ± 13 ms compared to *T_PVC_* = 197 ± 15 ms of propagated PVCs. Furthermore, spontaneous arrhythmias were detected in 17% simulations with 7 of these episodes being sustained.

### C. Propagated *versus* blocked PVCs

**FIG. 3.**
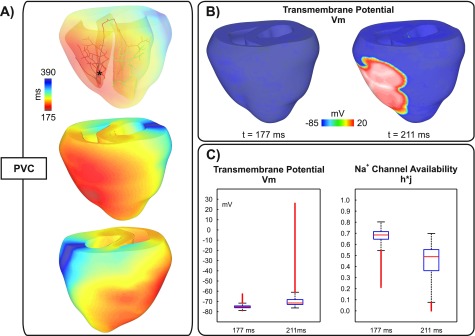
Propagated PVC originating in the HPS (asterisk) in a simulation with a negative-shift in the steady-state Na^+^ channel inactivation. (a) Activation sequence; (b) *V_m_* distributions in the BiV model at time instants: t1 = 177 ms, the time of the PVC, and t2 = 211 ms; (c) Box plots illustrating the distribution of *V_m_* and h*j at instants t1 and t2.

**FIG. 4.**
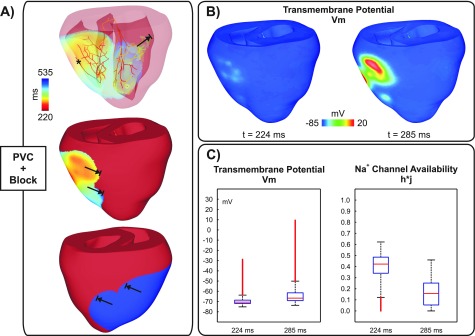
Conduction block of a PVC originating in the HPS (asterisk) in a simulation with a negative-shift in the steady-state Na^+^ channel inactivation. (a) Activation sequence; (b) *V_m_* distributions in the BiV model at time instants: t1 = 224 ms, the time of the PVC, and t2 = 285 ms; (c) Box plots illustrating the distribution of *V_m_* and h*j at t1 and t2.

Figures 3(a) and 4(a) show activation sequences illustrating two different outcomes of the stochastic BiV model with a negative-shift in the steady-state Na^+^ channel inactivation: a PVC that propagated throughout the ventricles; and a PVC that propagated into the RV, but failed to activate the LV. Although both PVCs originated in the Purkinje fibers of the RV, the foci in all N = 100 experiments were distributed throughout the HPS without any obvious preference. PVCs arising in the Purkinje fibers of the LV also led to conduction block and arrhythmias (not shown).

The PVC in Fig. 3 (see also the supplementary material, Movie 1) arose at *T_PVC_* = 177 ms and rapidly propagated to the neighboring fibers and to the RV. A second and a third PVC also originated in the HPS of LV and RV (locations not shown) at later time instants t = 220 ms and t = 234 ms, respectively. The average activation time of all PVJs in the RV was t = 211 ± 24 ms, while the PVJs in the LV activated later at an average t = 260 ± 14 ms. *V_m_* distributions in the BiV at times t = 177 ms (*T_PVC_*) and t = 211 ms (average PVJ activation in the RV) are shown in Fig. 3(b) whilst Fig. 3(c) presents overall *V_m_* elevations and Na^+^ channel availability h*j at the same time instants. Although average h*j in the ventricles was 0.4 ± 0.2 at t = 211 ms, there was not enough Na^+^ current available for successful wavefront propagation.

Block of the PVC in Fig. 4 was due to subthreshold DADs that inactivated the fast *I_Na_* current. The PVC originated at t = 224 ms, 47 ms later than the PVC in Fig. 3 (see the supplementary material, Movie 2 for further details). At this time, SCR-mediated DADs caused sufficient ventricular depolarization [Fig. 4(b)] to drive h*j to 0.4 ± 0.2 as can be seen in Fig. 4(c). All PVJs in the RV activated with an average t = 285 ± 24 ms initiating propagation into the RV. A later PVC (t = 282 ms - not shown) emerged in the HPS of the LV and also activated the posterior-apical region of the LV. However, wavefronts arriving in the LV at such later times (t = 285 ms) failed to propagate due to a large reduction in h*j, 0.2 ± 0.1, caused by SCR-mediated depolarizations [see Figs. 4(b) and 4(c)].

### D. SCR-mediated spontaneous arrhythmias

**FIG. 5.**
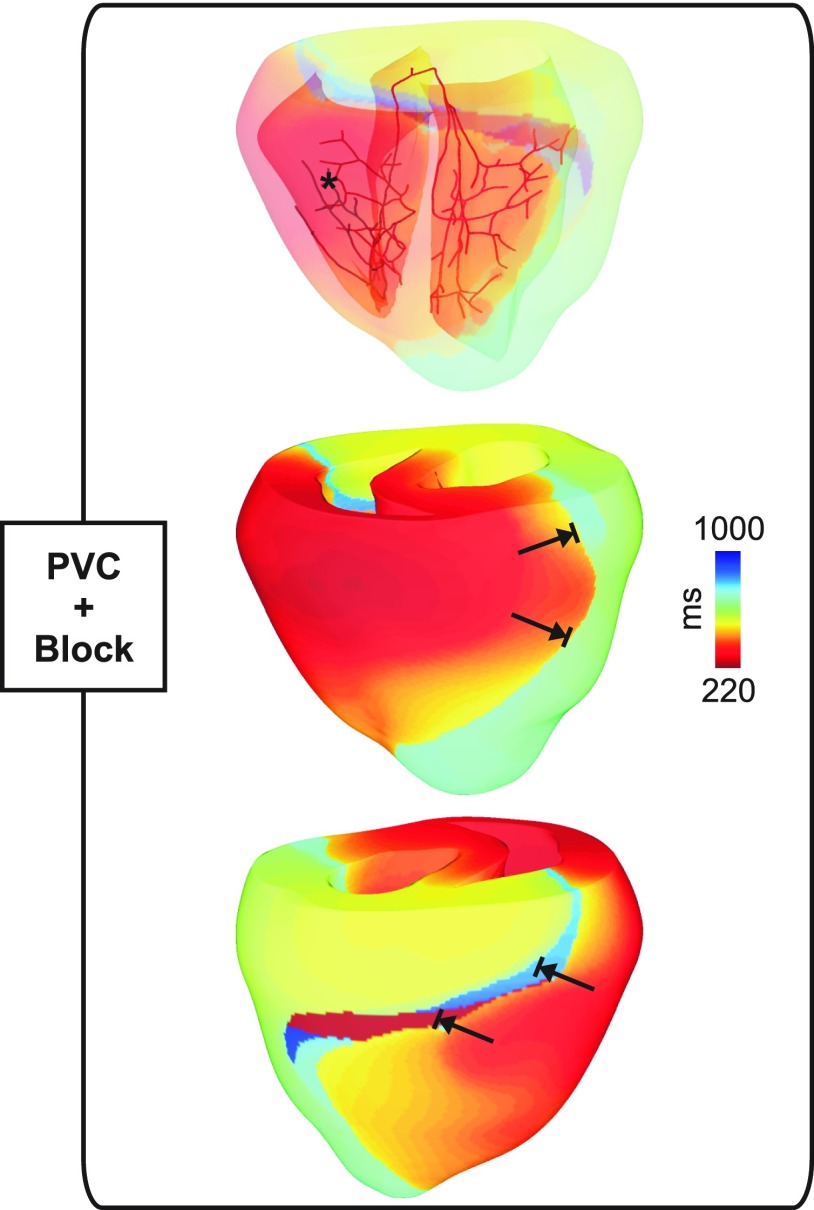
Activation sequence of a PVC originating in the HPS (asterisk) that blocks the LV in a simulation with a negative-shift in the steady-state Na^+^ channel inactivation.

**FIG. 6.**
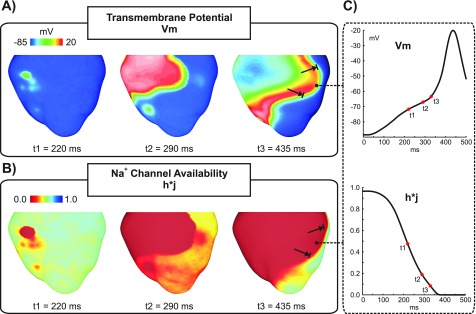
Conduction block of a PVC. (a) *V_m_* distributions in the BiV model at time instants: t1 = 219 ms, the time of the PVC; t2 = 290 ms, when the wavefront is advancing into the LV; and t3 = 435 ms, time of conduction block in the LV. (b) h*j maps at the same time instants as in (a). (c) *V_m_* and h*j traces at the location indicated by an asterisk in (a) and (b).

**FIG. 7.**
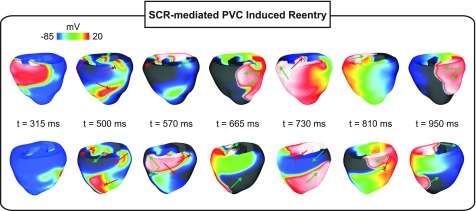
Arrhythmia induction following a SCR-mediated PVC in a simulation with a negative-shift in the steady-state Na^+^ channel inactivation. *V_m_* maps showing sustained arrhythmia induction by a SCR-mediated PVC originating in the HPS. Green arrows: successful wavefront propagation; red arrows: conduction block.

An example of how SCR-mediated PVCs can degenerate into a reentry is shown in Figs. 5–7 and supplementary material, Movie 3. A PVC emerging in the HPS (Fig. 5) at *T_PVC_* = 219 ms propagates to the RV and enters the LV, but is blocked at a later time instant in the LV free wall. A distinct line of block in the LV extending from the base to the apex can be seen in Fig. 5. Average PVJ activation was 261 ± 21 ms in the RV and 304 ± 16 ms in the LV. Similar to the PVC in Fig. 4, block was due to insufficient Na^+^ current necessary to overcome the electrotonic load posed by the 3D myocardium. This is illustrated in Fig. 6 with *V_m_* and h*j shown at different time instants. Note that DADs in the ventricles at the time of the PVC reduced overall h*j, but reduced Na^+^ channel availability was more pronounced in areas near PVJs since these cells were more severely depolarized due to (1) local SCR-mediated DADs; and (2) current flowing from the HPS which was also undergoing SCR-mediated DADs. At time t = 290 ms, the triggered PVC leaves the RV and enters the LV encountering myocytes with reduced h*j. At time t = 435 ms, block occurs in the LV due to insufficient Na^+^ current. Figure 6(c) presents *V_m_* and h*j transients in a cell located at the line of conduction block. Note the slow *V_m_* depolarization due to long lasting DADs and the consequent reduction in h*j. At t = 290 ms h*j was about 0.2 and was further reduced to ≈ 0.1 at the arrival time of the wavefront.

Figure 7 shows *V_m_* maps at different time instants throughout the reentry episode initiated by the PVC in Fig. 5. The wavefront triggered by the PVC traveled at a slower velocity in the septum due to reduced Na^+^ current (green arrow at t = 500 ms). At t = 570 ms, the wavefront attempts to reenter both the LVs, where it is initially blocked, and the RV. However, it blocks in the RV as well as towards the apex of the LV (red arrows) since the myocardium in those regions is still refractory. See the supplementary material, Movie 3 for further details. Note that at time instants t = 665 ms and t = 730 ms, the wavefront travels from the anterior part of LV to the RV (upper panel) and reenters the LV from the apex towards the base (lower panel) where the cells recover excitability. The heterogeneous block of wavefronts traveling towards and away from the base in the posterior part of the LV is due to the apicobasal gradient in APD: 266 ms in isolated endocardial cells from the base compared to 311 ms in the apex. At time t = 810 ms, the wavefront reenters the LV and proceeds to the RV, but is blocked towards the apex because apical cells are still in the ERP (longer APD). The wavefront meanders and reenters the LV from the apex at t = 950 ms in a similar way as at t = 665 ms. This reentry was sustained for >2000 ms (see the supplementary material, Movie 3).

## IV. DISCUSSION

In this study, we sought to determine the conditions under which PVCs resulting from stochastic subcellular SCR events can lead to ventricular arrhythmias, specifically, how Na^+^ current reduction can accentuate the occurrence of subthreshold DADs in the 3D myocardium causing conduction block of PVCs and reentry. To achieve this goal, a stochastic subcellular-scale mathematical model of SCR events was incorporated into an anatomically realistic model of the rabbit ventricles and HPS. Simulations with *I_Na_* reduction due to a negative-shift in the steady-state channel inactivation had a major impact on arrhythmia induction than *g_Na_* decrease. Electrotonic load modulated SCR-mediated DADs favoring PVC formation in the 1D fibers of the HPS and conduction block and reentry in the 3D myocardium. However, reentry induction was only observed in the presence of intrinsic electrophysiological heterogeneities in AP, otherwise in their absence no reentrant episodes were observed.

### A. Electrophysiological heterogeneities and DAD-triggered activity

Electrophysiological differences in ionic currents underlying the AP in cardiac myocytes have been reported in the ventricles.^28^ As a result, an APD gradient forms within the ventricular walls with endocardial myocytes having longer APDs than epicardial ones^38^ (see Fig. 1). Moreover, cells from the HPS differ in numerous ways from ventricular myocytes since their main role is rapid AP propagation to ensure a synchronized ventricular contraction.^27,39^ Such heterogeneities may render different regions of the heart more vulnerable to PVC formation.^40^ Unlike wet-lab experiments with intact animal hearts, our multiscale computational model allowed us to directly assess the influence of electrophysiological heterogeneities in SCR-mediated triggered arrhythmias by conducting simulations with two versions of the BiV model: a homogeneous model with the same cell model assigned to both HPS and ventricles; and a heterogeneous model (Fig. 1). Significant differences in terms of SCR-mediated PVC formation between both models were observed in two set of experiments: severe *g_Na_* reduction (25% to the control value in Table I) and shifting Na^+^ channel steady-state inactivation by −5 mV. In these particular sets of experiments, SCR-mediated DADs led to PVC formation in all simulations with the heterogeneous BiV model while causing subthreshold DADs in simulations with the homogeneous model. PVC formation in the heterogeneous model was favored by differences in ionic currents that underlie the AP of Purkinje cells in our model. DADs result from a delicate balance between depolarizing and repolarizing currents. In comparison to ventricular myocytes, Purkinje cells have more Na^+^ and less K^+^ currents which makes them more vulnerable to DADs.^40^ These intrinsic differences influence conduction velocity and APD,^27^ and are accounted for in our modified MSH model by increasing *g_Na_* and reducing *g_Kr_* and *g_Ks_* (see Table I).

### B. Electrotonic modulation of supra- and subthreshold DADs

Current experimental evidence shows that a variety of arrhythmias in structurally healthy ventricles is attributed to ectopic focal sources.^8,41^ Is well established that DADs in isolated myocytes are mainly caused by *I_NCX_* activity in response to SCR events.^4^ This mechanism is reproduced by our phenomenological model of SCR, as illustrated in Fig. 2. In cardiac tissue, on the other hand, the factors determining how DADs summate to trigger PVCs are still a topic of intense research.^9,10,42^ According to the liminal length concept, the tissue around a focal source must be raised above a voltage threshold in order to initiate a propagated response.^43^ The size of the focal source will depend on the radius of electrotonic influence, which in turn is influenced by factors such as cell-to-cell coupling.^9^ Recently we have demonstrated, using the same multiscale model as in this study, that in SR Ca^2+^ overload (Ca_*SR*_ > 1500 *μ*mol/l), the probability of SCR release events is high (close to 100%) and the timing variability of these events is small. This ensures a sufficient degree of spatiotemporal coincidence of SCR-mediated DADs to drive the tissue gradually to the firing threshold.^10^ This finding is corroborated by experiments in normal rabbit hearts where *β*-adrenergic receptor stimulation caused spatiotemporal synchronization of SR Ca^2+^ overload and SCR-mediated PVC formation.^42^ Despite the differences in the simulation protocol between our previous and present studies, PVCs were observed in all experiments except the cases where *g_Na_* was reduced to 25% and the negative-shift was applied in the steady-state Na^+^ channel inactivation of cells in the homogeneous BiV model. In these experiments, DADs were not of sufficient magnitude to trigger a PVC due to electrotonic currents that smoothed out differences in *V_m_* across neighboring cells.

Furthermore, in both versions of the BiV model used in this study, most foci arose in the HPS (see Figs. 3–5). This is in line with our previous findings, where we have shown that the critical Ca_*SR*_ threshold for triggering a PVC is lower in the 1D HPS than in the 3D myocardium.^10^ Thus, the tissue geometry predisposes locations of lowered electrotonic load to SCR-mediated suprathreshold DADs while causing subthreshold DADs in regions where source-sink mismatch is more accentuated.

### C. Subthreshold DADs and conduction block

While the role of DADs in triggered arrhythmias is well known,^8,42^ the pro-arrhythmogenic effects of subthreshold DADs are not completely understood. On one hand, small DADs can facilitate conduction by moving *V_m_* close to the threshold of the fast Na^+^ current. Long lasting small DADs, on the other hand, can slow conduction as Na^+^ channels start to inactivate at elevated *V_m_* levels.^44^ In pathological conditions, such as the Brugada syndrome^16^ and ischemia^45^ where Na^+^ current is reduced, subthreshold DADs may cause conduction block. Indeed, elevations in resting *V_m_* values caused by hyperkalemia, one of the main components of ischemia, have been shown to initially increase conduction velocity, but this trend reverses as hyperkalemia worsens, eventually resulting in block.^46^ In a recent study, Liu and colleagues^14^ demonstrated in a computer modeling study that DADs can generate both the trigger and the substrate for arrhythmias. Moreover, they showed that conduction block becomes increasingly probable as Na^+^ channel availability is reduced.^40^ However, DADs in their study were induced artificially using an externally prescribed time dependence rather than by stochastic SCR events as in our study. Furthermore, electrotonic effects due to ventricular anatomy and wavefront curvature resulting from fiber arrangement in the 3D ventricles were neglected in their simplified 1D and 2D tissue models. In this study, we have employed a mathematical model that reproduces experimentally observed features of SCR events such as timing distribution of spontaneous Ca^2+^ and key features of Ca^2+^ waves emanating from these sparks.^25^ All simulations were conducted in an anatomically realistic model of the rabbit ventricles incorporating an anatomy-based HPS which allowed us to investigate the role of tissue geometry in reentry induced by SCR-mediated PVCs. Unlike the results of Liu *et al.*^14^ reentry was not observed in a homogeneous model. Only the heterogeneous BiV model with severe *g_Na_* reduction (25% of its control value) or a left-shift in Na^+^ channel inactivation promoted conduction block and reentry. This result suggests that PVCs can be usually benign in healthy individuals, but under Na^+^ channelopathies they can precipitate life-threatening arrhythmias. In fact, the latter representation of the Na^+^ current loss-of-function mimics physiologically conditions associated with LQT3 and Brugada syndromes,^30,31^ inherited diseases with an increased risk of sudden cardiac death caused by ventricular arrhythmias. In our simulations, block was caused by reduction in Na^+^ channel availability due to long lasting subthreshold DADs as depicted in the inset of Fig. 2(b). Indeed, block of PVCs such as those in Figs. 4 and 5 were observed in experiments where average *T_PVC_* was > 200 ms in contrast to propagated PVCs (Fig. 3) which were triggered earlier.

Although PVCs in Figs. 4(a) and ^5^ were triggered at similar time instants (224 and 219 ms, respectively), the extent of block was different. The PVC in Fig. 4 propagated into the RV, but failed to activate the LV whereas the PVC in Fig. 5 resulted in a heterogeneous conduction block in the LV. Block of the PVC in Fig. 4(a) is the combined result of late triggering and slowed conduction. Partial inactivation of Na^+^ channels causes a slowing of conduction velocity.^47^ Thus, the slower the velocity of the activation wavefront, the longer cells undergoing subthreshold DADs remain at elevated *V_m_* values which further inactivates Na^+^ channels. Differences in conduction velocity between both simulations are translated to the activation times of PVJs in the RV: 285 ms ± 24 ms for the PVC in Fig. 4(a) compared to 261 ± 21 ms for the PVC shown in Fig. 5. Epicardial breakthrough of the PVC in Fig. 5 can be seen close to the base of the RV at t1 = 219 ms in Fig. 6(a). The wavefront spread then at a slow velocity over the thin-walled RV towards the thick-walled junction with septum and LV (t2 = 290 ms) where propagation was blocked at time instant t3 = 435 ms. Interestingly, reduced Na^+^ current was shown to create a substrate for conduction block induced by ectopic stimuli near regions of tissue expansion such as the RV outflow tract.^13^ By contrast, in our study conduction block occurred due to a large reduction in Na^+^ channel availability. *V_m_* and h*j transients [Fig. 6(c)] from a location adjacent to the line of block illustrate this mechanism. SCR-mediated DADs caused tissue depolarization, but electrotonic currents prevented the initiation of a propagated AP. At the time of wavefront arrival t3, most of Na^+^ channels were in an inactivated state which resulted in block of the PVC in that region.

### D. Importance of heterogeneity in SCR-mediated arrhythmias

Intrinsic electrophysiological heterogeneities within the ventricular wall are believed to be arrhythmogenic since they can cause non-uniform repolarization and conduction block.^48^ The role of such differences in AP morphology and ERP was assessed by comparing the susceptibility of homogeneous and heterogeneous BiV models to arrhythmia induction by SCR-mediated PVCs. Reentry was observed only in the heterogeneous BiV model. Specifically, reentry occurred in 2% and 17% of experiments with *g_Na_* reduced to 25% and a negative-shift in the steady-state Na^+^ channel inactivation, respectively. Interestingly, using a similar heterogeneous BiV computational model of the rabbit ventricles, Bishop *et al.*^19^ demonstrated that differences in APD resulted in arrhythmia initiation following premature paced beats. Consistent with their findings, our study shows that the transmural differences in ionic currents result in complex activation/repolarization patterns and heterogeneous conduction block. This mechanism is evident in Fig. 7. Myocytes from the RV at time t = 570 ms are still refractory, thus causing a block in that region. Cells from the basal part of LV, on the other hand, had recovered excitability, allowing the wavefront to reenter. Furthermore, Bishop *et al.* concluded that gradients in APD, particularly in the apicobasal direction, play an important role in the formation of heterogeneous conduction block.^19^ Our findings are in line with their result. Wavefronts at times t = 570 ms and t = 810 ms in Fig. 7 (lower panel) were also blocked in the posterior part of the LV when traveling from the base to the apex where APD is longer. The PVCs then curl and return to the LV from the apex towards the base (see the lower panel, times t = 665 ms and t = 950 ms).

### E. Limitations

Several limitations of this study should be mentioned. First, the abnormal RyR function is a key pathophysiological mechanism in HF^49^ and CPVT.^8^ Ca^2+^ overload may not play a significant role in PVC formation in such conditions. However, in the phenomenological model used in this study SCR events have only a functional dependence on Ca_*SR*_, which is adjusted based on experimental measurements of SCR statistics in cardiac myocytes.^25,37^ Nevertheless, in scenarios associated with the malfunction of RyR channels, the threshold level for SCR events is lowered and the amount of Ca^2+^ required to provoke SCR events is reduced. Second, although a specific model of the AP in Purkinje cells exists,^27^ we used the augmented MSH model^26^ to describe Ca^2+^ cycling and voltage dynamics in both ventricles and HPS. Here, our goal was to investigate how PVCs resulting from stochastic SCR events can lead to arrhythmias. The phenomenological model used in this study accounts for the experimentally observed features of SCR events and could readily be coupled to the MSH model.^25^ Nevertheless, ionic currents of the MSH model were modified to represent the main electrophysiological properties of Purkinje fibers.^27^ Moreover, myocytes with a distinct APD morphology and duration have also been identified in ventricles.^50^ These cells give rise to isolated islands of APD prolongation between the endocardial and epicardial layers, but their exact distribution within the ventricular wall is still not known. Thus, in this study, we followed previous modeling approaches^19,20^ and considered the midmyocardium as an anatomically discrete myocyte population with distinct electrophysiological properties.^51^ However, in a recent study, Walton and colleagues^52^ demonstrated that electrotonic interactions across PVJs are sufficient to modulate APD locally in the myocardium. By including a layer of specialized midmyocardium, we could directly assess the arrhythmogenic influence of functional heterogeneities. Effects of different configurations regarding the position, thickness, and topography of midmyocardial cells are out of the scope of this work.

Furthermore, Na^+^ channelopathies are modeled here by simply reducing *g_Na_* or shifting Na^+^ channel steady-state inactivation by −5 mV. However, the electrophysiological mechanisms of cardiac diseases associated with *I_Na_* loss-of-function, such as the Brugada syndrome, are highly heterogeneous with disease-related arrhythmias linked either to depolarization or repolarization disorders.^16,53,54^ The deliberate modeling choice in the design of the BiV model allowed us to dissect out the consequences of *I_Na_* reduction on arrhythmia induction by SCR-mediated PVCs. By using the exact same cellular modifications, other electrophysiological abnormalities thought to play a role in disease-related arrhythmias in Brugada and LQT3 syndromes could be ruled out. Finally, our findings are specific to the rabbit ventricles and further investigation is necessary to draw conclusions related to the role of electrophysiological heterogeneity in SCR-mediated arrhythmias in humans. However, rabbits are relevant animal models of human cardiac electrophysiology due their known similarities of ion channel distribution with the human heart and suitability for repolarization and arrhythmia studies.^55^ Furthermore, simulations of the electrical activity in the rabbit ventricles has a computationally cheaper cost due to its smaller size.

## V. CONCLUSIONS

This study makes use of a mathematical model of SCR events coupled to an anatomically accurate model of rabbit ventricles and HPS to investigate the origin of triggered arrhythmias. Our biophysically detailed simulations revealed that electrophysiological heterogeneities in ionic currents and a specific case of Na^+^ channel loss-of-function can lead to reentry induced by SCR-mediated PVCs. SCR events led to PV formation in the HPS since DADs are more likely to reach threshold within their 1D fibers. In the 3D myocardium, on the other hand, SCR events caused subthreshold DADs due to the greater electrotonic load imposed on the cells. These subthreshold DADs resulted in heterogeneous conduction block and reentry in myocytes with a negative shift in Na^+^ channel steady-state inactivation. From a clinical standpoint, these findings suggest that PVCs, while benign in healthy individuals, can precipitate life-threatening ventricular arrhythmias when combined with Na^+^ channelopathies associated with ischemia, HF, LQT3, and Brugada syndromes. Furthermore, Na^+^ channel blockers (Class I antiarrhythmic drugs) may pose a pro-arrhythmic risk to patients suffering from conditions promoting SCR events such as HF and CPVT.

## VI. SUPPLEMENTARY MATERIAL

See the supplementary material for Movie 1: genesis of an SCR-mediated PVC in the HPS. Purkinje (left superior), epicardial basal (right superior), epicardial anterior (left inferior), and epicardial posterior (right inferior) views of *V_m_* maps on the surface of the ventricles (same scale as Fig. 3); Movie 2: conduction block of a SCR-mediated PVC originating in the HPS in a simulation with a negative-shift in steady-state Na^+^ channel inactivation. Purkinje (left superior), epicardial basal (right superior), epicardial anterior (left inferior), and epicardial posterior (right inferior) views of *V_m_* maps on the surface of the ventricles (same scale as Fig. 4); and Movie 3 arrhythmia induction following an SCR-mediated PVC in the HPS. Purkinje (left superior), epicardial basal (right superior), epicardial anterior (left inferior), and epicardial posterior (right inferior) views of *V_m_* maps on the surface of the ventricles (same scale as Fig. 7).
